# An Open-Source 3D Printed Three-Fingered Robotic Gripper for Adaptable and Effective Grasping

**DOI:** 10.3390/biomimetics10010026

**Published:** 2025-01-04

**Authors:** Francisco Yumbla, Emiliano Quinones Yumbla, Erick Mendoza, Cristobal Lara, Javier Pagalo, Efraín Terán, Redhwan Algabri, Myeongyun Doh, Tuan Luong, Hyungpil Moon

**Affiliations:** 1 Facultad de Ingeniería en Mecanica y Ciencias de la Producción, Escuela Superior Politecnica del Litoral, ESPOL, Campus Gustavo Galindo, Km. 30.5 Vía Perimetral, Guayaquil 090902, Ecuador; eridamen@espol.edu.ec (E.M.); crilver@espol.edu.ec (C.L.); gpagalo@espol.edu.ec (J.P.); eaterac@espol.edu.ec (E.T.); 2 Department of Engineering Technologies, University of Puerto Rico, 2100 Ave. Sur, Carolina 00984, Puerto Rico; emiliano.quinones@upr.edu; 3 Research Institute of Engineering and Technology, Hanyang University, Asan 15588, Republic of Korea; redhwanalgabri@gmail.com; 4 Department of Mechanical Engineering, Sungkyunkwan University, 2066 Seobu-ro, Jangan-gu, Suwon 16419, Republic of Korea; ehauddbs@g.skku.edu (M.D.); luongtuan@g.skku.edu (T.L.); hyungpil@g.skku.edu (H.M.)

**Keywords:** adaptive gripper, additive manufacturing, force sensor, robotic arm

## Abstract

This research focuses on the design of a three-finger adaptive gripper using additive manufacturing and electromechanical actuators, with the purpose of providing a low-cost, efficient, and reliable solution for easy integration with any robot arm for industrial and research purposes. During the development phase, 3D printing materials were employed in the gripper’s design, with Polylactic Acid (PLA) filament used for the rigid mechanical components and Thermoplastic Polyurethane (TPU) for the flexible membranes that distribute pressure to the resistive force sensors. Stress analysis and simulations were conducted to evaluate the performance of the components under load and to gradually refine the design of the adaptive gripper. It was ensured that the mechanism could integrate effectively with the robotic arm and be precisely controlled through a PID controller. Furthermore, the availability of spare parts in the local market was considered essential to guarantee easy and cost-effective maintenance. Tests were conducted on an actual robotic arm, and the designed gripper was able to effectively grasp objects such as a soda can and a pencil. The results demonstrated that the adaptive gripper successfully achieved various types of grasping, offering a scalable and economical solution that represents a significant contribution to the field of robotic manipulation in industrial applications.

## 1. Introduction

The human hand is one of nature’s most intricate and versatile manipulation systems, capable of grasping, manipulating, and sensing objects with remarkable dexterity. Its adaptability has been essential to human evolution and development. In robotics, engineers and scientists have sought to emulate and surpass the capabilities of the human hand by developing advanced gripping systems [1].

Robotic grippers, typically positioned at the end of a robotic arm’s kinematic chain, serve as the primary interface for interacting with objects, similar to the human hand. Robotic grippers play a crucial role in both industrial and scientific applications by handling a diverse array of objects with varying materials, sizes and shapes [2]. Unlike the human hand, grippers can be customized for environments and tasks beyond human reach. Performing repetitive high-speed actions, lifting heavy loads and operating in extreme conditions are the principal motivators for the design and continuous study of grippers [3,4].

Over the past few decades, grippers have evolved across various industries, where they are now used for complex tasks across multiple fields [5]. For instance, in automotive manufacturing, grippers manipulate large vehicle components, requiring designs with high load capacities and large metal structures [6,7]. In the food industry, grippers execute pick-and-place operations for product handling [8,9,10]. The medical industry relies on grippers for tasks involving precise handling of samples and equipment [11]. However, some tasks, such as manipulating wet or porous objects, still require manual intervention due to the gripping complexity [8,12].

Agricultural applications, in particular, impose specific requirements on gripper design, such as the ability to adapt to irregular shapes and to apply precise gripping forces that avoid damaging delicate produce [13]. This degree of adaptability often necessitates high degrees of freedom and integrated sensors, though these improvements can significantly raise costs [14].

Given the wide range of objects that require manipulation, grippers must be able to perform multiple gripping modes to effectively handle various tasks and meet the changing demands of the industry [15]. Effective gripping mechanisms are essential for stability during tasks [3]. Adaptive grippers, which adjust to object shapes, achieve this through design choices and adjustments in gripping modes [16]. However, adding adaptability to traditional grippers can introduce design complexity and high costs, which limits widespread adoption.

To further enhance adaptability, touch sensors and vision systems are increasingly integrated into grippers. These additions, combined with coordinated joint control, allow rigid structures to achieve adaptability by incorporating additional degrees of freedom and precise position control of each joint [17]. Both fully constrained kinematic chains and less-constrained chains with damped couplings can provide a balance between adaptability and control [18].

Despite their utility, adaptive grippers are often costly, which limits their widespread use. Most industrial grippers today are two-finger designs, which are simple to manufacture and reliable for a range of standard tasks [5,19]. However, as industries increasingly require handling objects of diverse shapes, two-finger designs cannot meet these demands. The need for adaptable gripping solutions has motivated the development of more sophisticated adaptive grippers, with three-finger configurations emerging to provide enhanced dexterity and control, however, at a higher cost [20].

Adaptive grippers can yield significant benefits, including improved efficiency, productivity, and safety, making them a valuable investment in industrial applications [5]. Nevertheless, their high costs limit widespread adoption and create a demand for low-cost adaptive solutions [20].

In the context of affordable designs, several studies have developed 3D-printed gripping manipulators with fixed gripping postures, including two-finger grippers [21,22,23], three-finger grippers [24,25,26,27,28,29,30] and four-finger gripper [31]. Although these designs have improved the accessibility of affordable manipulators, their fixed gripping configurations limit adaptability to objects of varying shapes and sizes [18].

**Figure 1:**
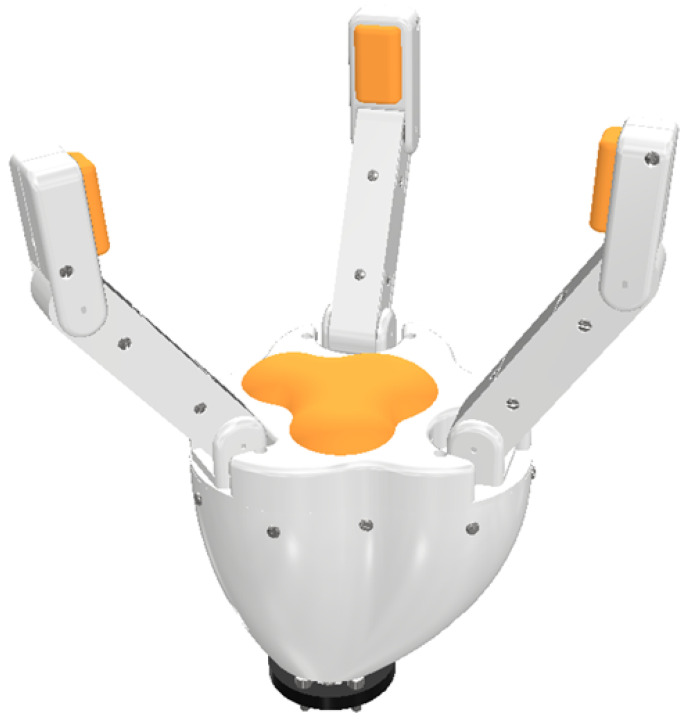
Open-source 3D Printed Three-Fingered Robotic Gripper.

This project addresses this need by designing an accessible, low-cost adaptive gripper compatible with an ABB industrial robotic arm, as shown in Figure 1. The proposed gripper offers the adaptability to perform three types of gripping modes (cylindrical, parallel, and spherical) critical for versatile object handling [18]. By leveraging 3D printing, a widely accessible and affordable manufacturing method, this project aims to create a three-fingered adaptive gripper that can meet industry needs without the associated high costs, and that is accessible to the general public.

## 2. Adaptive Three-Finger Gripper with Rigid Links

This section describes the functional requirements, proposed solution, mechanical and electrical design, control architecture, and prototype development of the proposed adaptive three-fingered gripper. Complete information on the design is organized in a Github repository (https://github.com/RAMEL-ESPOL/Three-Fingered-Robotic-Gripper Access date: January 2025).

### 2.1. Functional Requirements and Conceptual Design

The gripper design must meet the following requirements:

- **Adaptability**: Ability of the finger to adopt different positions through the movement of its joints.
- **Cost**: The design must remain low-cost, with accessible components.
- **Efficient Power Transmission**: The chosen mechanism must provide minimal energy loss from the motor shaft to the joint during the torque transmission process.
- **Space optimization**: The chosen mechanism must be able to fit within the structure of the finger, occupying the least amount of space.
- **Precision**: The chosen mechanism should enable precise joint rotation control for accurate gripping.

**Figure 2:**
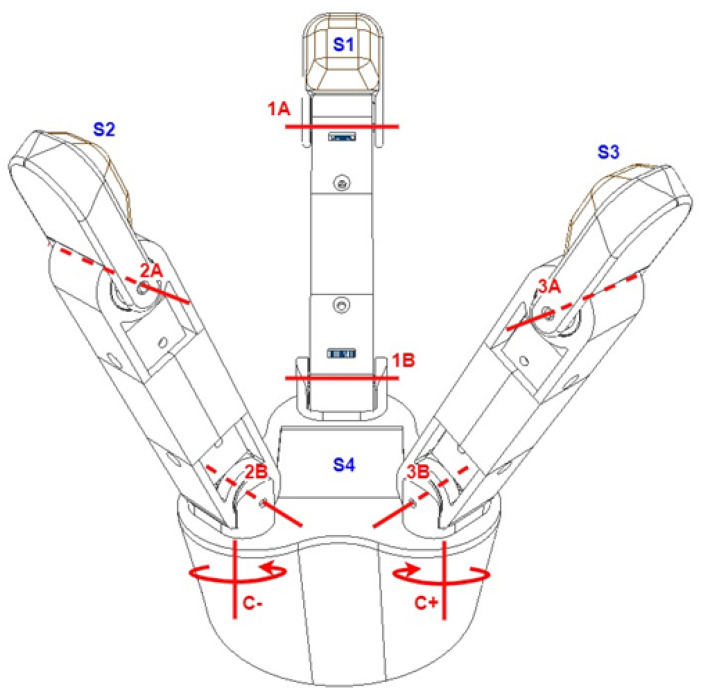
Diagram of the 3-finger robotic gripper kinematic structure. The central finger (S1) operates with two revolute joints (1A and 1B), allowing it to move similarly to a 2-joint planar articulated robot. The outer fingers (S2 and S3) also feature two revolute joints each (2A, 2B and 3A, 3B), along with additional joints (C+ and C−) that allow rotation around their base.

Figure 2 illustrates an initial concept design of the proposed solution, highlighting the primary elements. The conceptual design consists of a palm and three fingers, which provides greater adaptability than the typical two-finger design. The gripper includes four sensors: three on the fingertips (S1, S2, S3) for force detection and one on the gripper palm (S4) for pressure detection.

Each finger has two rotational axes (A and B) controlled by two independent motors, providing at least two degrees of freedom (DOF) per finger and thus facilitating varied grip positions and enhanced adaptability. In addition to each finger’s two DOFs, the design introduces an extra DOF by allowing two fingers to rotate inward and outward. This rotation, indicated as C+ and C− in Figure 2, is achieved using an internal servo motor and a gear mechanism that ensures synchronized but opposing rotations.

To improve assembly, maintenance, and part replacement, the design emphasizes modularity. Each component can be developed individually, supporting a flexible and durable gripper structure.

### 2.2. Design Development

**Figure 3:**
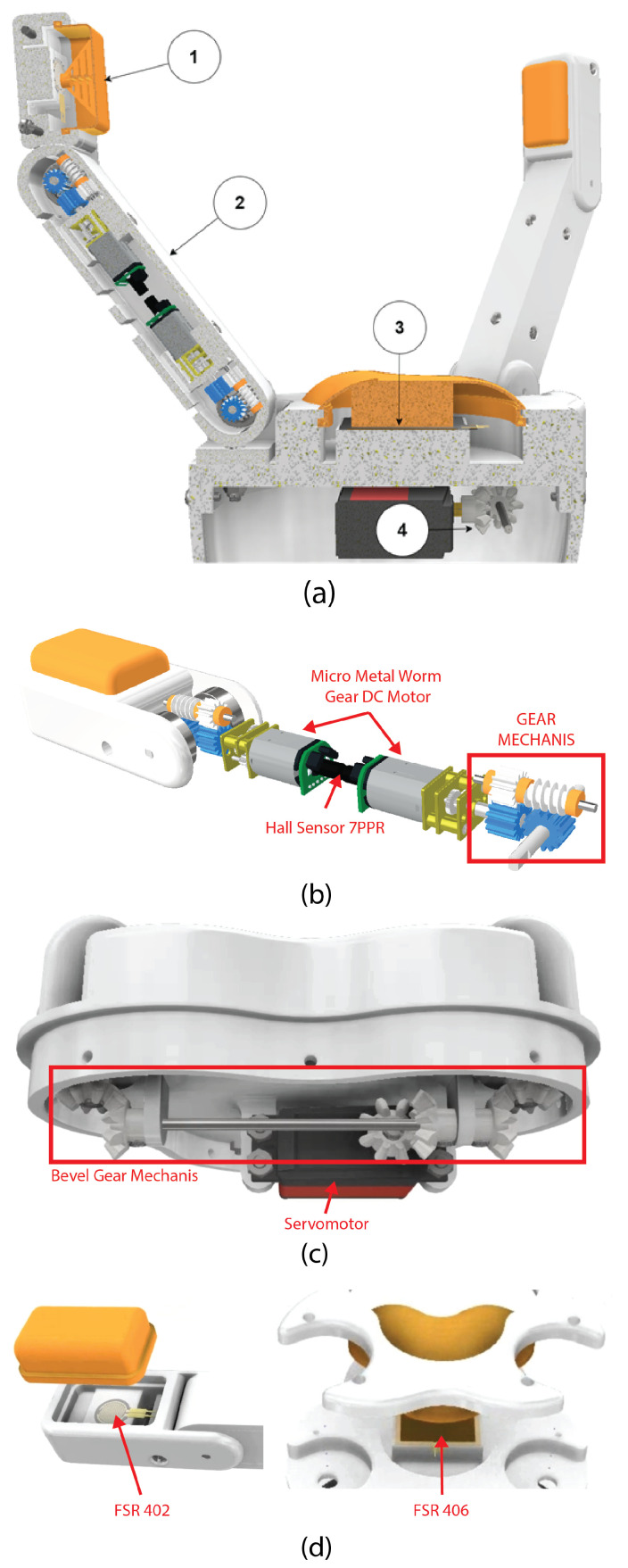
Diagram of the Three-Fingered Gripper design. Half-cut view of the gripper (**a**), showing its main components: the finger rotational mechanism (**b**), where direct current (DC) micro motors control the rotation of the thumb and intermediate links (0° to 135°) using a gear mechanism and Hall effect sensors for precise movement; the bevel gear system (**c**), which enables the outer fingers to rotate around their own axes (0° to 90°); and the flexible membranes (**d**), which distribute pressure to the force sensors (FSR 402, FSR 406) through linear spaces that allow material bending.

The adaptive three-fingered gripper, illustrated in Figure 3, represents the 3D design of the gripper with rigid links. The prototype includes two key materials for manufacturing the components: flexible TPU membranes (shown in orange) and rigid PLA parts, both fabricated through 3D printing using an FDM (Fused Deposition Modeling) technique.

The TPU membranes were printed with a layer height of 0.2 mm and a low infill density of 10% to maintain flexibility, while ensuring sufficient tensile strength to distribute pressure across the force-sensing resistors (FSR). For the rigid PLA parts, a layer height of 0.16 mm was selected to enhance precision, with an infill density of 50% to improve structural integrity.

In Figure 3a, Parts 1 and 3 incorporate flexible TPU membranes to evenly distribute pressure on the FSR. This was accomplished by incorporating a grid-like pattern during the modeling process and adjusting the slicing software settings to ensure optimal bending performance. while Part 3 achieves flexibility in the palm region through reduced infill density during printing. Part 2 contains the internal mechanism for rotating the finger joints’ axes illustrated in Figure 2. Finally, Part 4 includes the mechanism that allows two fingers to rotate around their base, connected via bevel gears to a shared main shaft. To ensure the precision of these interlocking components, supports were added during the printing process, and the parts underwent post-processing to remove residual material and ensure accurate fitting and functionality.

Figure 2 depicts the spatial arrangement of each finger in the gripper, positioned 120° apart and evenly spaced on an imaginary circular plane. For modularity, each finger follows a uniform design from the base of rotation to the distal end.

The first Micro Metal Worm Gear motor (axis A) in Figure 3b drives rotation of the final link (thumb), where the force sensor is located. The second motor (axis B) controls the rotation of the intermediate link that connects the final link to the palm base. Both motors have a movement range between 0° and 135°. These motion ranges are calibrated to meet the demands of various grip types, including flat, cylindrical, and spherical. For increased adaptability, the servomotor in Figure 3c uses a bevel gear system to facilitate rotation of two fingers around their axes in parallel but opposite directions, with a designated range of 0° to 90°.

Two approaches were evaluated for transmitting movement from the motors to the finger joints. The first option involved aligning the motor parallel to the joint axis for direct rotation without additional gearing. The second option positioned the motor perpendicularly to the joint axis to optimize link space. For this reason, the chosen approach utilizes straight and worm gears available in the local market, for a 90° torque transmission from the motor shaft to the link axis, as demonstrated in Figure 3c. Finally, we prototype the gripper with the specification in Table 1.

### 2.3. Stress Analysis

**Figure 4:**
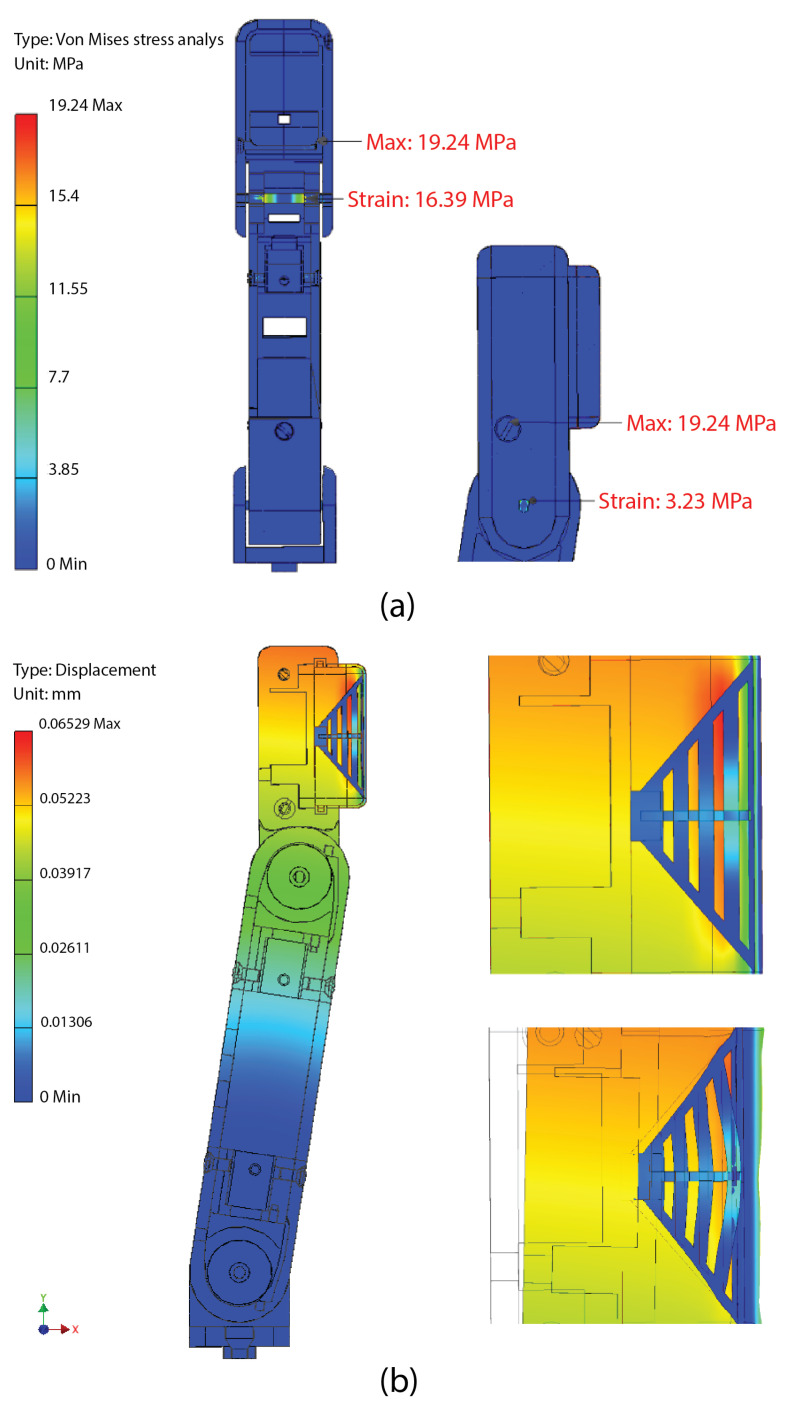
Stress analysis performed on one of the fingers of the gripper using Autodesk Inventor: (**a**) Von Mises stress analysis of a fingertip under maximum motor torque. (**b**) Deformation analysis of the fingertip’s TPU membrane. The design and patterns of the TPU membrane of the fingers must be able to perform a smooth and precise grip on objects.

Figure 4a presents the stress analysis performed on one of the fingers of the gripper using Autodesk Inventor. For this analysis, the maximum allowable torque from the selected motor (196 N·mm) was applied on the axis of the external phalanx responsible for pressing the objects with the fingertips. The materials used for the simulation were acrylonitrile butadiene styrene (ABS) for the rigid links, offering similar properties to the PLA used in prototyping, and rubber for the elastic membrane, simulating the mechanical characteristics of TPU. The yield strength for ABS and the stainless steel used in the shaft are approximately 32 MPa and 200 MPa, respectively. The maximum Von Mises stress observed remained well within these material thresholds, indicating a sufficient safety margin.

Additionally, Figure 4b depicts a deformation analysis for the TPU fingertip membrane. The bottom illustration highlights the membrane’s strain under load at the fingertip, where the resistive force sensor is positioned. The analysis demonstrates that the force applied is effectively distributed across the membrane, concentrating the pressure precisely at the sensor’s contact point.

### 2.4. Force Analysis

**Figure 5:**
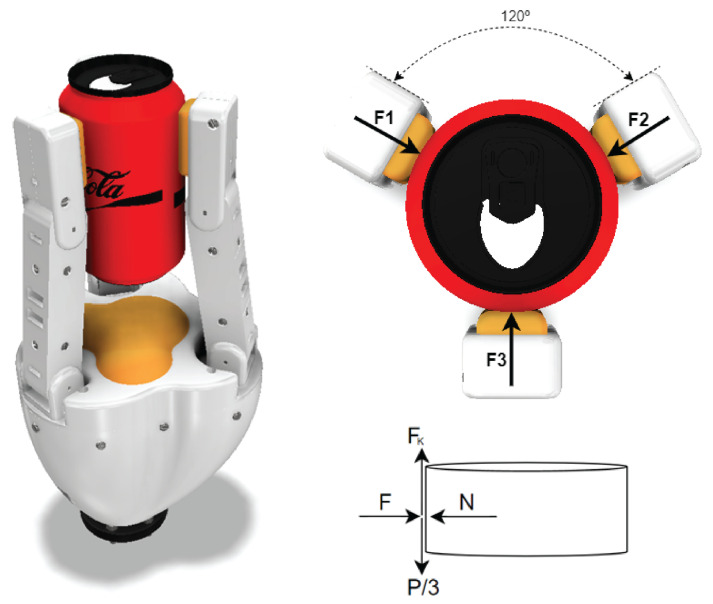
Force diagram at a point of contact, showing a top view, an isometric view, and the free body diagram (FBD) of the contact point. The forces F1, F2, and F3 act tangentially with the object and the friction between the finger membrane and the object surface, producing a stable and precise grip.

To select the appropriate motors capable of providing the required rotational torque, the necessary force to achieve a minimum grip range was analyzed, considering the weight of the objects to be gripped. Figure 5 illustrates a cylindrical grip, where the load is equally distributed across the three fingertips, effectively reducing the load on each finger to 1/3 of the total object weight. The free-body diagram in Figure 5 reflects this distribution, with the following force relationship:

(1) |F1|=|F2|=|F3|=|F|

For calculating the required motor torque, a static friction coefficient of $\mu_{s}=0.3$ was applied. This value, based on ABS material properties, provides a conservative baseline, as the final gripper material will be TPU, which generally offers a higher friction coefficient when printed using additive manufacturing. This conservative approach establishes a safety factor, ensuring that the selected motors can handle higher torque if needed.

The static friction coefficient influenced by material interaction, in this case aluminum and ABS, was selected based on the ASTM D1894 test method, which indicates that the corresponding coefficient is the lowest value among both materials. Aluminum typically presents a coefficient above 0.4 in self-contact tests, though a precise value for TPU and aluminum would require specific testing under our design conditions.

For the mass of the cylindrical object, a base weight of 500 g was chosen to account for variations, slightly exceeding the approximate weight of a standard 350 mL soda can (378 g). This increase provides an approximate safety margin of 32%, ensuring that the gripper can securely handle objects of similar dimensions and weights.

### 2.5. Hardware

**Figure 6:**
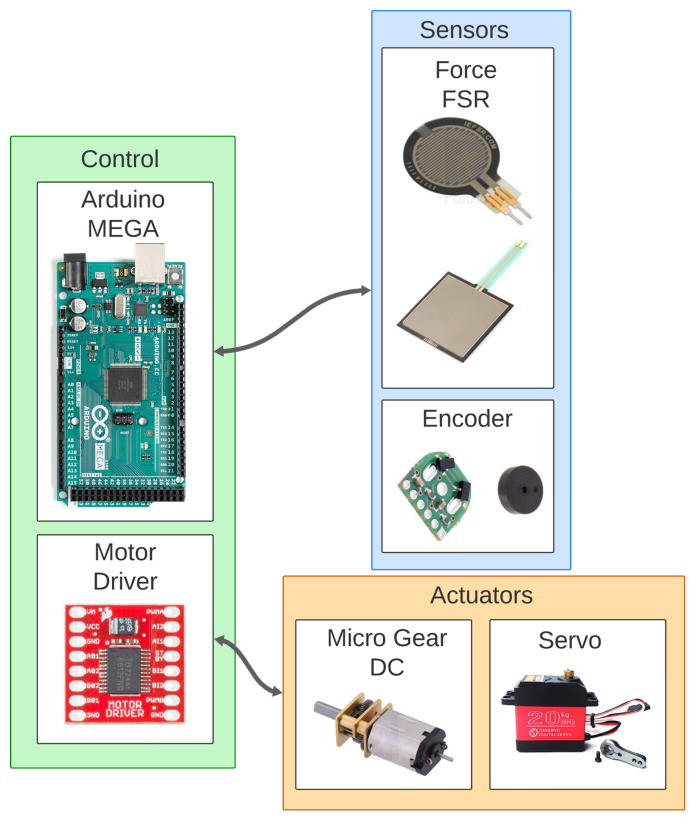
Diagram of the gripper system hardware, showing controllers, sensors, and actuators.

The system hardware design includes actuator and electronic component selection. The control system schematic, show in Figure 6, includes an Arduino Mega, six Micro Metal Worm Gear motors with encoders, corresponding motor drivers, a servomotor, and resistive force sensors.

The Arduino serves as the system controller, managing component operations and processing sensor data. Motor drivers amplify Arduino signals to efficiently power and control the six motors, enabling independent speed and direction adjustments. The servomotor adjusts the gripper’s positions, controlled via PWM signals. The resistive force sensors, a device that vary its resistance based on deformation, send analog signals to the Arduino, allowing it to process force feedback from the gripper. Due to the motors’ substantial current requirements, the system is powered by a dedicated 5V – 3A supply separate from the Arduino electronics, reducing interference and ensuring stable operation.

The selected hardware components and their functionalities are detailed below:

- Arduino^®^ MEGA: is a microcontroller board based on the ATmega2560 (Arduino, Ivrea, Italy). It has 54 digital input/output pins (of which 15 can be used as PWM outputs), 16 analog inputs, 4 UARTs (hardware serial ports) and an ICSP header (In-Circuit Serial Programming for programming microcontrollers).
- Servomotor, DS3218 High Torque Metal Gear Digital Servo (DSME, Tianjin, China): The DS3218 servomotor is a high-torque, metal-gear digital servo with a water-resistant design. It provides 20 kg of torque with a 270-degree range of rotation, making it suitable for handling the gripper’s position adjustments under varying loads.
- Dual Motor Driver, TB6612FNG (Toshiba Semiconductor and Storage, Kawasaki, Japan): The TB6612FNG dual motor driver allows independent control of two bidirectional DC motors or one bipolar stepper motor. It supports a motor voltage of 4.5 V to 13.5 V and a peak current output of 3 A per channel (1 A continuous), making it a good choice for the low-power motors used in this design.
- Micro Metal Gearmotor: This gearmotor is a compact, high-power 12 V brushed DC motor equipped with long-lasting carbon brushes and a metal gearbox with a gear ratio of 4.995:1. Its small cross-section (10 × 12 mm) and extended 9 mm output shaft with a 3 mm diameter make it ideal for precise, space-efficient applications.
- Magnetic Encoder: this kit that uses a magnetic disc and Hall effect sensors provides 20 counts per revolution of the motor shaft. The sensors operate from 2.7 V to 18 V and provide digital outputs that can be connected directly to a microcontroller, enhancing positional feedback accuracy.
- Force-sensing resistors (FSR), 1.5 cm-Diameter Circle FSR and 4 × 4 cm Square FSR: These FSRs from Interlink Electronics are passive components that exhibit decreased resistance in response to increased force applied to their active areas. The FSRs provide force feedback across different surface areas, suitable for measuring applied force on different parts of the gripper’s contact surfaces.

### 2.6. Control

**Figure 7:**
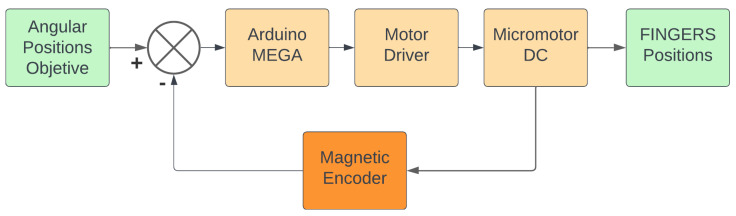
Gripper closed-loop control system block diagram.

A control circuit was implemented in the motors of the joints of each finger to control the grip, which takes advantage of the hall effect sensors to measure the angular position of the joint axis. The motor speed values were regulated through a PID control, which, through the Arduino, stops or compensates the energy received by the motor until a specific angular position is reached. The block diagram of the process can be seen in Figure 7. The PID control parameters implemented in the system ($K_{P}$, $K_{I}$, $K_{D}$) were obtained using the Ziegler Nichols method. Due to the nature of the gripper’s functions, no overshooting was desired in the system. Therefore, the value of the ultimate gain and the oscillation period of the system was determined by increasing $K_{P}$ and bringing the $K_{D}$ and $K_{I}$ values to zero. The values of the remaining profits were determined using the Ziegler Nichols equations. Since the system varied the state of the motors through electronic drivers that controlled the speed, the system gains are based on a PD control to determine $K_{D}$, and then through the iterative manual method, the value of $K_{I}$ is determined. The resulting values are $K_{P}$: 1.5, $K_{I}$: 0.005 and $K_{D}$: 0.24375.

**Figure 8:**
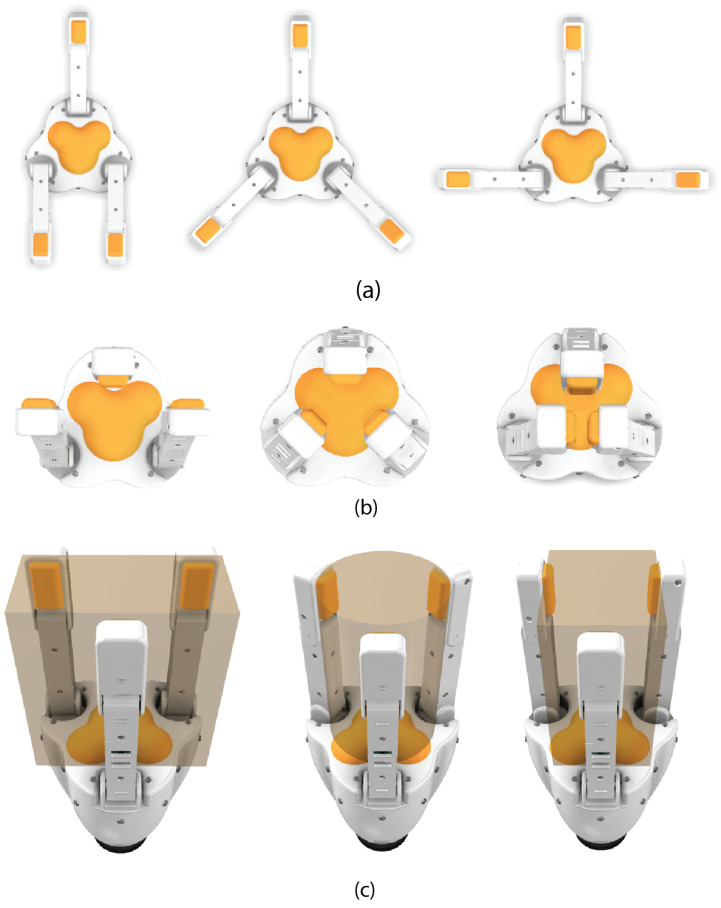
Grip positions: (**a**) open in three basic grip configurations (flat, cylindrical-spherical and tangential-flat), (**b**) closed gripper in base configurations, and (**c**) maximum working volume for each contact grip configuration at the base of the palm.

In addition, the posture of the gripper fingers is controlled by a servomotor through the Arduino. This enables the capability to perform various types of prehension. Figure 8 shows the different types of grip prior to their final implementation. A positioning simulation of the gripper was carried out in three different configurations that include a flat grip, cylindrical or spherical grip, and pincer-type grip with an extra finger for additional support, as seen in Figure 8a,b. Subsequently, the working volumes corresponding to each type of grip in the designed gripper were extracted, as seen in Figure 8c. In the flat grip, there is a width of 70 mm (between the planes of the membranes), a length of 140 mm, and a standard height of 150 mm, which will be the same in the three types of grip. The cylindrical-spherical grip, has a 100 mm diameter with the same height. Lastly, the tangential-flat grip features a rectangular-type working volume with a length of 85 mm (between the planes of the two pincer fingers), a width of 100 mm (towards the plane of the membrane of the extra finger), and the same 150 mm height. Additionally, it is taken into consideration that the object’s height must be approximately 150 mm to align with the palm base sensor and fingertip sensors to ensure proper contact. To adapt to different objects, each gripping style accommodates slight variations in object height. Likewise, it is possible to grip objects of greater length and width, constrained by the maximum distance between the axes of the middle phalanx, as long as there is no need to make contact with the palm’s base sensor.

## 3. Simulation and Prototype Test

**Figure 9:**
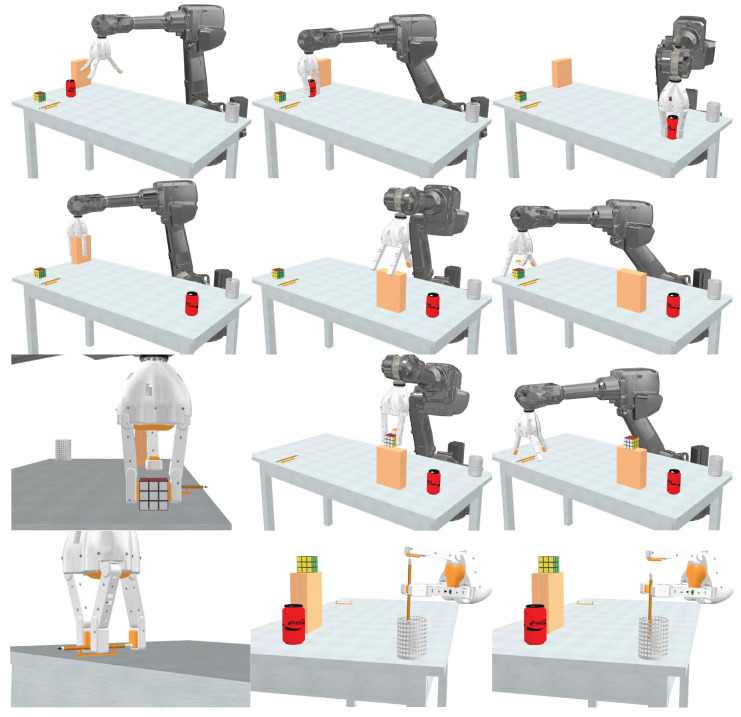
Simulation in Autodesk Inventor showing the gripper transporting various objects (a soda can, Rubik’s cube, box, and pencil) between different positions over a table.

A prototype of the proposed solution was implemented, with the specifications in Table 1, after a simulation of its integration was carried out, with an ABB brand robotic arm of the IRB 2600 type (12 kg payload, and 1.65 m height). Figure 9 provides screenshots of the simulation carried out, where various objects are manipulated, validating the three types of grips allowed by the design. For the following figures presented, emphasis is placed on the three grip configurations previously proposed and their interaction with objects. In Figure 9 left column, the gripper can be seen in an open position, highlighting its ability to adapt to objects of various sizes.

**Figure 10:**
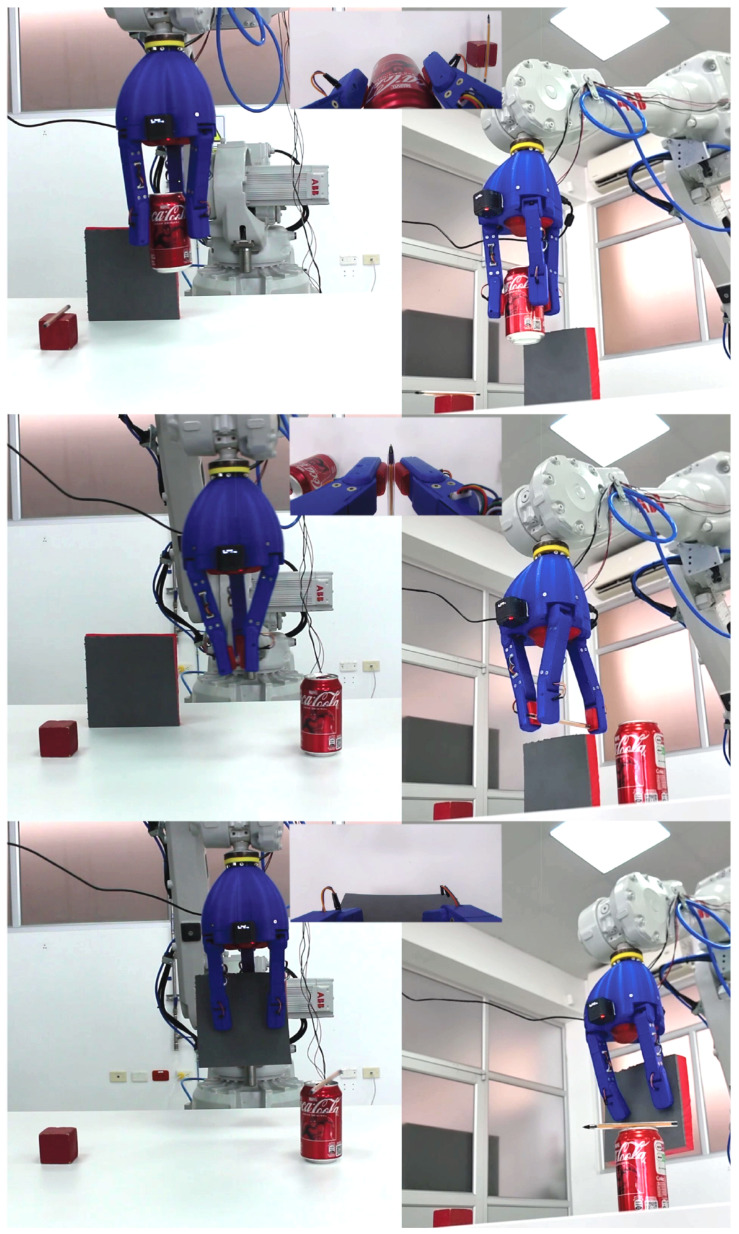
Real environment pictures of a gripper mounted on an ABB IRB2600 industrial robot, displaying three gripping configurations: flat, cylindrical-spherical, and pincer. In the last row the gripper is also holding a soda can and a pencil for a verification test Screenshots from a video showing the ABB IRB2600 industrial robot using the gripper. The images illustrate the gripper performing three gripping actions: picking up a soda can, a pencil, and a rectangular object, corresponding to the cylindrical, pincer, and flat grips, respectively.

Figure 10 highlights the gripper in action, demonstrating its ability to handle a variety of objects. The images show the gripper successfully picking up a soda can, a pencil, and a rectangular object, illustrating its adaptability to different shapes and sizes. These actions correspond to the cylindrical, pincer, and flat grips, respectively. The interaction between the flexible membranes at the fingertips and the objects activates the FSR sensors, ensuring precise control. With a resistance of 10 kOhm in the sensor circuit, forces from 0.98 N to 98 N were manually recorded, verifying the sensors’ functionality within the required force range exerted by the internal actuators.

## 4. Summary and Conclusions

This paper presents the design, analysis, and validation of a versatile, low-cost adaptive gripper intended for integration with the ABB IRB2600 industrial robotic arm. The objective of designing a three-finger adaptive gripper prototype has been successfully achieved through the use of additive manufacturing and electromechanical actuators. This solution has not only proven to be an economical alternative to industrial traditional gripper systems but has also demonstrated high functionality and adaptability. The use of additive manufacturing allowed continuous mechanical design improvement by facilitating proofs of concept without resorting to high prototyping costs. Also, the implementation of electromechanical actuators made it possible to adjust the degrees of freedom of the gripper, giving it greater mobility and adaptability proportional to the number of actuators used. The combination of these technologies, along with the control electronics, has enabled the creation of a versatile gripper that can be efficiently integrated with an ABB robotic arm. The system requires the computer to act as a communication bridge between the gripper and the robotic arm, defining the grasping states for the evaluated objects.

Concerning the designed gripper contact points, a printed TPU membrane was generated with specific parameters and internal structure, so an accurate interaction with the resistive force sensors was obtained, which required a correct distribution of the force applied on its contact surface. In its implementation, it was proven that a detection range of 0.1 0.98 N up to 98 N force is achieved, which demonstrates that the selection of established sensors allows full control of the force of contact with objects within the range that they can provide the actuators.

The control system developed using Arduino MEGA has enabled precise and reliable manipulation of the gripper. This was possible through the implementation of a Proportional-Integral-Derivative (PID) controller for adjustment of the joint’s angular position, which takes advantage of the feedback provided by the encoders of the DC micromotors. As a result, different grasping postures were evaluated with and without objects, including cylindrical, flat, and pincer grasps. To increase its reliability, resistive sensors were incorporated into the gripper fingers. These sensors can measure the amount of force applied to the surface of the fingers, thus ensuring the integrity of both the gripper and the objects handled. Ultimately, this adaptive gripper prototype exemplifies how innovative engineering can drive significant advances in industrial automation and the evolution of robotics toward greater availability and adaptability in changing environments.

## Figures and Tables

**Table 1:** Three-Fingered Robotic Gripper specifications.

Number of Fingers	Three (3)
Degrees of Freedom	3 fingers by 2 motors = 6 + 1 rotate
Actuation	Type: DC MicroMotor
	Gear Ration: 1:236
	Max Torque: 0.2 (Nm)
	Max. Joint Speed: 12 (RPM)
Weight	Finger: 0.14 kg
	Palm: 0.81 kg
	All: 1.23 kg
Joint Resolution	Encoder: 7 PPR
Communication	TTL serial data
Payload	3 kg
Power Requirement	12 and 5 VDC 3A

## Data Availability

Data are contained within the article.
